# Spotlight on the Binding Affinity of Ion Channels for Phosphoinositides: From the Study of Sperm Flagellum

**DOI:** 10.3389/fphys.2022.834180

**Published:** 2022-02-07

**Authors:** Takafumi Kawai, Yasushi Okamura

**Affiliations:** ^1^Integrative Physiology Program, Graduate School of Medicine, Osaka University, Suita, Japan; ^2^Graduate School of Frontier Bioscience, Osaka University, Suita, Japan

**Keywords:** phosphoinositides, ion channel, voltage-sensing phosphatase, sperm flagellum, Slo3, KCNQ

## Abstract

The previous studies revealed that many types of ion channels have sensitivity to PtdIns(4,5)P_2_, which has been mainly shown using heterologous expression system. On the other hand, there remains few evidence showing that PtdIns(4,5)P_2_ natively regulate the ion channel activities in physiological context. Our group recently discovered that a sperm specific K^+^ channel, Slo3, is natively regulated by PtdIns(4,5)P_2_ in sperm flagellum. Very interestingly, a principal piece, to which Slo3 specifically localized, had extremely low density of PtdIns(4,5)P_2_ compared to the regular cell plasma membrane. Furthermore, our studies and the previous ones also revealed that Slo3 had much stronger PtdIns(4,5)P_2_ affinity than KCNQ2/3 channels, which are widely regulated by endogenous PtdIns(4,5)P_2_ in neurons. Thus, the high-PtdIns(4,5)P_2_ affinity of Slo3 is well-adapted to the specialized PtdIns(4,5)P_2_ environment in the principal piece. This study sheds light on the relationship between PtdIns(4,5)P_2_-affinity of ion channels and their PtdIns(4,5)P_2_ environment in native cells. We discuss the current understanding about PtdIns(4,5)P_2_ affinity of diverse ion channels and their possible regulatory mechanism in native cellular environment.

## Introduction

**FIGURE 1:**
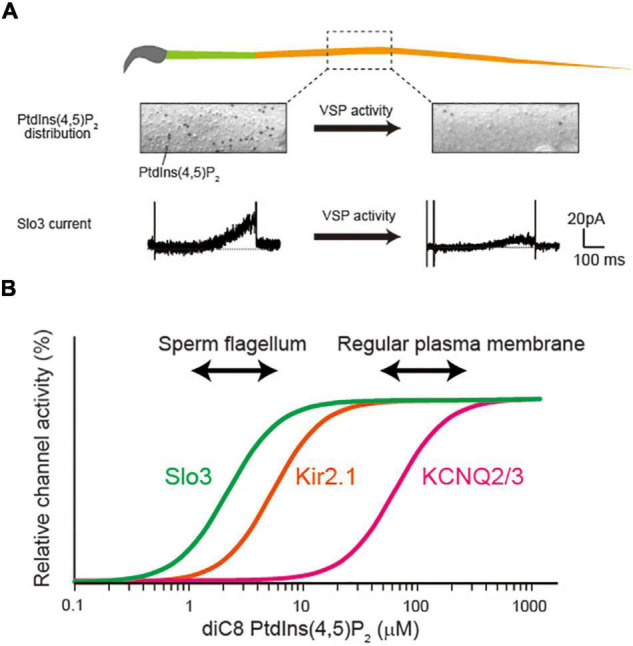
Possible relationship between PtdIns(4,5)P_2_ concentration and its affinity for ion channels. **(A)** Voltage-sensing phosphatase (VSP) reduces the PtdIns(4,5)P_2_ levels in sperm flagellum, regulating Slo3 activity. The figure is illustrated based on the finding in our previous report (Kawai et al., 2019). The PtdIns(4,5)P_2_ is detected by freeze-fracture electron microscopy. The VSP changes the distribution of PtdIns(4,5)P_2_ levels and regulates Slo3 activity. The Slo3 activity was measured by perforated patch clamp recording (Kawai et al., 2019). **(B)** Diagram showing the different PtdIns(4,5)P_2_ dependency among different ion channels.

Phosphoinositides (PIPs) comprise a minor proportion of the lipid membrane, but they play important roles in a variety of physiological processes, including signal transduction, regulation of cytoskeleton, exocytosis, and endocytosis (Balla, 2013). Specifically, PtdIns(4,5)P_2_, one class of PIPs, mainly exists in the inner leaflet of the plasma membrane. Accumulating evidence suggest that PtdIns(4,5)P_2_ also regulates the property of diverse ion channels in many aspects (Suh and Hille, 2005, 2008; Okamura et al., 2018). For example, PtdIns(4,5)P_2_ is required for the basal activities of all five members of voltage-gated potassium KCNQ/Kv7 channels (Kv7.1-7.5 or KCNQ1-5) (Zhang et al., 2003). Currently, more than 50 ion channel molecules are identified as the targets of PtdIns(4,5)P_2_ regulation from the electrophysiological studies using heterologous expression system (Suh and Hille, 2008; Okamura et al., 2018). In these studies, the PtdIdns(4,5)P_2_ dependency of ion channels are examined using application of soluble short chain PtdIdns(4,5)P_2_, activation of voltage-sensing phosphatase (VSP), or Gq-couple receptors activation. In contrast to the plenty of knowledge about ion channel regulation by PtdIns(4,5)P_2_ from these experiments, the number of reports about these regulatory mechanisms is quite limited in native physiological condition. The evidence of its physiological importance originates in the study of M-current in sympathetic neurons (Brown and Adams, 1980), which is now widely observed in various types of neurons. The M-current is evoked by the stimulation of muscarinic acetylcholine receptors (mAchRs), which activates GPCR/Gq signaling cascade and reduces the PtdIns(4,5)P_2_ level in plasma membrane. The reduced PtdIns(4,5)P_2_ level causes the suppression of KCNQ2/3 channel activities, inducing the depolarization of neurons. The observation of M-current in native neurons indicates that KCNQ channels is constitutively activated by PtdIns(4,5)P_2_ in normal condition, and the change of PtdIns(4,5)P_2_ level is also physiologically important for the neural function. Our group recently reported that Slo3, a sperm specific K^+^ channel, is natively regulated by PtdIns(4,5)P_2_ in sperm flagellum (Kawai et al., 2019). We analyzed the function of VSP, which dephosphorylates PtdIns(4,5)P_2_ into PtdIns(4)P, in a mouse spermatozoa. We found that the amount of PtdIns(4,5)P_2_ was significantly and highly upregulated in a VSP-deficient spermatozoa, thereby the activity of Slo3 was significantly increased (Figure 1A; Kawai et al., 2019), which is consistent with the previous report that Slo3 is sensitive to PtdIns(4,5)P_2_ (Tang et al., 2010). Importantly, we observed that the principal piece, to which Slo3 specifically localizes, showed extremely low density of PtdIns(4,5)P_2_ compared with the previously reported regular plasma membrane (Kawai et al., 2019). The Slo3 has strong binding affinity for PtdIns(4,5)P_2_ and the low level of PtdIns(4,5)P_2_ appears to adjust to the high-affinity of Slo3 for proper regulation (Figure 1B). The previous reports indicate that Slo3 has more than 10-fold higher affinity for PtdIns(4,5)P_2_ than KCNQ2/3 channels and that the PtdIns(4,5)P_2_ level in principal piece is also less than one-tenth of the regular plasma membrane as reported in fibroblast (Fujita et al., 2009). Thus, the study sheds light on the importance of focusing on the relationship between PtdIns(4,5)P_2_-affinity of ion channels and the native PtdIns(4,5)P_2_ level. Nevertheless, we sometimes discuss the PtdIns(4,5)P_2_-sensitivity of ion channels in an all-or-none manner, and the affinity of PtdIns(4,5)P_2_ is not so importantly discussed. In the present mini-review, we describe how the PtdIns(4,5)P_2_ sensitivity or affinity of ion channels has been examined in heterologous expression system at first. Then, we introduce the list of PtdIns(4,5)P_2_-sensitive ion channels with their affinity as described. Finally, we discuss the possibility that different PtdIns(4,5)P_2_-affinity of ion channels are regulated in different PtdIns(4,5)P_2_ environment of specialized subcellular compartments.

### Quantifying the Affinity of Ion Channels for PtdIns(4,5)P_2_ Using Heterologous Expression System

The PtdIns(4,5)P_2_-sensitivity of ion channels is also mostly examined by electrophysiology. The ion channel of interest is heterologously expressed in *Xenopus* oocyte or other cell lines such as Human Embryonic Kidney cells 293 (HEK-293) or Chinese hamster ovary (CHO) cells. There are several techniques to manipulate plasma membrane PtdIns(4,5)P_2_ levels so that the change in ion channel activities is monitored. For example, heterologous expression of Gq-couple receptors such as muscarinic acetylcholine receptor M1 is widely used for this purpose. The agonist stimulation cleaves PtdIns(4,5)P_2_ to reduce its level on the plasma membrane. Furthermore, a rapamycin-inducible phosphatase, Pseudojanin, strongly depletes PtdIns(4,5)P_2_, as well as PtdIns(4)P (Hammond et al., 2012). Although these chemical techniques are important molecular tools to manipulate the PtdIns(4,5)P_2_, it may not be suited for estimating PtdIns(4,5)P_2_-affinity of ion channel due to the difficulty in precisely manipulating the PtdIns(4,5)P_2_ levels.

The most quantitative method to analyze PtdIns(4,5)P_2_–affinity is performed by directly applying a soluble short acyl chain PtdIns(4,5)P_2_ to the inner leaflet of plasma membrane in an inside-out configuration. This method allows to describe a dose-response curve and to calculate the Kd-value, suited for discussing the PtdIns(4,5)P_2_-affinity of ion channels. On the other hand, because this technique uses short acyl chain PtdIns(4,5)P_2_ instead of native long acyl chain lipids, it may show some artificial effects on ion channel properties. Indeed, although the PtdIns(4,5)P_2_ sensitivity of several K^+^ channels has been already reported by perfusing soluble PtdIns(4,5)P_2_, the sensitivity has not been reproduced using other techniques to deplete PtdIns(4,5)P_2_ (Kruse et al., 2012). A possible alternative way to estimate PtdIns(4,5)P_2_–affinity is using VSP, which dephosphorylates PtdIns(4,5)P_2_ in response to the depolarization (Murata et al., 2005). Because VSP depletes the endogenous PtdIns(4,5)P_2_ by its enzyme activity, it can examine the importance of the regulation by PtdIns(4,5)P_2_ in a more physiological aspect. Although the quantitativity of VSP for PtdIns(4,5)P_2_–affinity is not more precise than that of soluble PtdIns(4,5)P_2_ perfusion, the strength of VSP activity can be easily controlled by changing the amplitude, duration, and/or number of the depolarization pulses, conferring a certain level of quantitativity to it. Ideally, it should be the best way to quantify the PtdIns(4,5)P_2_-affinity of ion channels by soluble PtdIns(4,5)P_2_ perfusion and confirm the result using VSP experiments.

### Difference in the PtdIns(4,5)P_2_ –Affinity Among Ion Channels

As described above, more than 50 ion channel molecules have already been shown to be PtdIns(4,5)P_2_ -sensitive (Suh and Hille, 2005, 2008; Okamura et al., 2018), including K^+^ channels, Ca^2+^ channels, TRP channels, P2X receptors, NMDA receptors, and so on. Some studies also performed the detailed experiments and estimated the Kd value of PtdIns(4,5)P_2_–affinity by perfusing a short acyl chain PtdIns(4,5)P_2_. We listed the representative examples of the PtdIns(4,5)P_2_ affinity of ion channels (Table 1). As shown in Table 1, the Kd value for PtdIns(4,5)P_2_ sensitivity of ion channels has large variation, ranging from 100 nM to 100 μM. The PtdIns(4,5)P_2_-affinities of ion channels themselves may also change depending on the states of channel activity. For example, TRPM8, which is activated by both cold and menthol, changes their apparent PtdIns(4,5)P_2_ affinity in response to the agonistic stimuli; cold and menthol (Rohacs et al., 2005). Similarly, BK, Ca^2+^ activated-K^+^ channels (Tang et al., 2014), and TMEM16A, Ca^2+^ activated-Cl^–^ channels, also show different affinity to PtdIns(4,5)P_2_ depending on the intracellular Ca^2+^ concentration (Le et al., 2019).

**TABLE 1:** The PtdIns(4,5)P_2_ affinity of individual ion channels.

Name of the ion channel	Kd (μM)	References
Kv7.2/Kv7.3	∼87	Zhang et al., 2003; Li et al., 2005
Kir2.1	∼5	Lopes et al., 2002; Du et al., 2004
Kir2.3	∼29	Du et al., 2004; Ha et al., 2018
Kir3.4	17.7 ± 1.0	Jin et al., 2002
TREK-1	0.125	Chemin et al., 2005
Slo3	2.4 ± 0.3	Tang et al., 2010
BK	∼14 (0 [Ca^2+^]*_i_)* ∼6 (100 μM [Ca^2+^]*_i_*)	Tang et al., 2014
SK2	1.9 ± 0.22	Zhang et al., 2015
TRPV1	0.60 ± 0.2	Ufret-Vincenty et al., 2011
TRPV6	78.9 ± 6.5	Velisetty et al., 2016
TRPM2	11.9 ± 1.1	Barth et al., 2021
TRPM8	80.7 (25°C) 38.2 (17°C) 4.69 (17°C with 500 μM menthol)	Rohacs et al., 2005
TMEM16A	1.95 (0.5 μM [Ca^2+^]*_i_*)	Le et al., 2019
TMEM16F	5.8 ± 0.5	Ye et al., 2018
P2X7	20–30	Zhao et al., 2007
HCN	10.9	Pian et al., 2006

In most cases, PtdIns(4,5)P_2_ electrostatically interacts with positive charge residues, such as lysine and arginine, through its negative charge on the hydrophilic head group. However, the regulatory mechanism of ion channel activity by PtdIns(4,5)P_2_ is sometimes complicated and involves multiple sites of the ion channels. In KCNQ2/3, which is one of the most investigated ion channels about PtdIns(4,5)P_2_ regulation, the studies suggest that there are multiple interacting sites with PtdIns(4,5)P_2_; the interface of voltage sensor domain and pore-gate domain, the helix A-B linker site, and the distal C-terminus site (Zaydman and Cui, 2014). Recent cryo-EM structures of KCNQ channels with PtdIns(4,5)P_2_ also support the idea that PtdIns(4,5)P_2_ interacts with voltage sensor and pore-gate domains (Sun and Mackinnon, 2017, 2020; Zheng et al., 2021). On the other hand, in Kir2.2, it is proposed that PtdIns(4,5)P_2_ binds at an interface between the transmembrane and the cytoplasmic domains, opening the inner helix gate (Hansen et al., 2011). The full-length protein structure of Slo3 has not been resolved so far, but the previous mutation analysis indicates that PtdIns(4,5)P_2_ binds to positive charge residues located at the linker region between pore-gate domain and cytosolic RCK1 domain (Tang et al., 2010). Thus, the regulatory mechanism by PtdIns(4,5)P_2_ of ion channels are diverse and sometimes so complicated, making it difficult to predict which residue is responsible for PtdIns(4,5)P_2_ sensitivity only from the 3D structure information. Such diverse regulatory mechanism of by PtdIns(4,5)P_2_ may partially explain the reason why the PtdIns(4,5)P_2_ affinities are totally different among different types of ion channels.

### Relationship Between PtdIns(4,5)P_2_ –Affinity of Ion Channels and the PtdIns(4,5)P_2_ Environment

How is such PtdIns(4,5)P_2_ affinity important for the regulation of native ion channel activities? In spite of many reports about PtdIns(4,5)P_2_ sensitivity in ion channels described above, there are only a few examples showing that the native ion channel activities are regulated by change of PtdIns(4,5)P_2_ level in physiological condition. The most representative example is the regulation of KCNQ2/3 activity by mAchR activation, which is known as M-current. Because KCNQ2/3 is constitutively activated by endogenous PtdIns(4,5)P_2_, the reduction of PtdIns(4,5)P_2_ closes the KCNQ2/3, inducing the depolarization of the cell. Another example was recently reported from our group (Kawai et al., 2019). We found that a sperm specific K^+^ channel, Slo3, which is also PtdIns(4,5)P_2_-sensitive (Tang et al., 2010), is regulated by VSP activity.

To understand the mechanism of ion channel regulation by native PtdIns(4,5)P_2_, it is important to consider the relationship between PtdIns(4,5)P_2_-affinity and membrane PtdIns(4,5)P_2_ concentration (Figure 1B). The affinity of KCNQ2/3 for PtdIns(4,5)P_2_ is reported to be rather low among PtdIns(4,5)P_2_-sensitive ion channels (Table 1). Importantly, the Kd value of KCNQ2/3 (∼87 μM) is supposed to be similar to the plasma membrane’s PtdIns(4,5)P_2_ level in regular cells (Figure 1B). On the other hand, the affinity of Slo3 for PtdIns(4,5)P_2_ (Kd = ∼2.5 μM) is more than 10-fold higher than that of KCNQ2/3 (Figure 1B). Interestingly, VSP generates biased distribution of PtdIns(4,5)P_2_ in sperm flagellum, optimizing the flagellar PtdIns(4,5)P_2_ environment for regulating a high-affinity Slo3 channel activity. Collectively, PtdIns(4,5)P_2_-affinity of ion channels must be in the range of physiological change for dynamic regulation of activity.

As shown in Table 1, a large proportion of ion channels have much stronger affinity for PtdIns(4,5)P_2_ than KCNQ2/3. This fact appears to indicate that only a minor population of PtdIns(4,5)P_2_-sensitive ion channels would be natively regulated by the change of PtdIns(4,5)P_2_ level in plasma membrane of regular cells. However, it is noteworthy that the distribution of PtdIns(4,5)P_2_ in plasma membrane is sometimes heterogeneous. The previous reports showed that PtdIns(4,5)P_2_ is concentrated at the rim of caveolae, a subset type of lipid raft, in cultured fibroblasts and smooth muscle cells of a mouse using freeze-fracture replica method (Fujita et al., 2009). Furthermore, it has been recently shown that there is compartmentalization of PtdIns(4,5)P_2_ metabolism; PtdIns(4,5)P_2_ break-down by G protein/PLC pathway is more rapid in cholesterol-rich domain (raft domain) than in cholesterol-poor domains (non-raft domain) of the plasma membrane (Myeong et al., 2021). These lines of evidence may suggest that lipid raft structure is more suited for regulating ion channel activities for PtdIns(4,5)P_2_ low-affinity ion channels due to the abundant PtdIns(4,5)P_2_ level.

The level of PtdIns(4,5)P_2_ can also be a variable depending on the subcellular compartments. For example, PtdIns(4,5)P_2_ is enriched in dendritic spines of cultured hippocampal neurons (Horne and Dell’acqua, 2007). The specialized structures such as flagellum or cilia can also have different PtdIns(4,5)P_2_ environment. As described above, we discovered that sperm flagellum show extremely low level of PtdIns(4,5)P_2_ due to VSP activity. Furthermore, several reports revealed that primary cilia, a non-motile single sensory organelle, show extremely low level of PtdIns(4,5)P_2_ due to accumulation of Inpp5e, a phosphoinositide 5-phosphatase (Chavez et al., 2015; Garcia-Gonzalo et al., 2015). Interestingly, this PtdIns(4,5)P_2_ level can also be dynamically regulated by the depletion of Inpp5e during cell-division cycle (Phua et al., 2017). Therefore, the activity of high PtdIns(4,5)P_2_ affinity ion channels, if there is any in primary cilia, could be regulated during the cell division cycles.

Overall, the different PtdIns(4,5)P_2_-affinity among ion channels may implicate that their regulation by PtdIns(4,5)P_2_ is dependent on the individual PtdIns(4,5)P_2_ environment, although it is also possible that PtdIns(4,5)P_2_-affinity have the other significance than regulation of ion channel activity, such as localization and trafficking of ion channels (Van Den Bogaart et al., 2011). In any case, it is important to focus on the PtdIns(4,5)P_2_-affinity, as well as the spatial information, to precisely examine the ion channel function in the future.

## Author Contributions

TK and YO wrote the draft of the manuscript and approved the submitted version.

## Conflict of Interest

The authors declare that the research was conducted in the absence of any commercial or financial relationships that could be construed as a potential conflict of interest.

## Publisher’s Note

All claims expressed in this article are solely those of the authors and do not necessarily represent those of their affiliated organizations, or those of the publisher, the editors and the reviewers. Any product that may be evaluated in this article, or claim that may be made by its manufacturer, is not guaranteed or endorsed by the publisher.
